# Characterization of four structurally diverse inhibitors of SUR2-containing K_ATP_ channels

**DOI:** 10.1080/19336950.2024.2398565

**Published:** 2024-09-20

**Authors:** Kangjun Li, Vaishali Satpute Janve, Jerod Denton

**Affiliations:** a Department of Pharmacology, Vanderbilt University, Nashville, TN; b Department of Anesthesiology, Vanderbilt University Medical Center, Nashville, TN; c Warren Center for Neuroscience Drug Discovery, Vanderbilt University, Nashville, TN

**Keywords:** Potassium channels, K_ATP_ channels, SUR2B, drug discovery, channelopathies, cardiovascular disease

## Abstract

Vascular smooth muscle ATP-sensitive potassium (K_ATP_) channels play critical roles in modulating vascular tone and thus represent important drug targets for diverse cardiovascular pathologies. Despite extensive research efforts spanning several decades, the search for selective inhibitors that can discriminate between vascular K_ATP_ (i.e. Kir6.1/SUR2B) and pancreatic and brain K_ATP_ (i.e. Kir6.2/SUR1) channels has, until recently, been unsuccessful. Our group therefore carried out a high-throughput screen of chemically diverse compounds with the goal of discovering specific Kir6.1/SUR2B inhibitors. This screen identified several novel classes of Kir6.1/SUR2B inhibitors, including the first potent (IC_50_ ~100 nM) and selective inhibitor published to date, termed VU0542270. Here, we expand on this work by disclosing the identity and pharmacological properties of four additional Kir6.1/SUR2B inhibitors that are structurally unrelated to Kir to VU0542270. These inhibitors, named VU0212387, VU0543336, VU0605768, and VU0544086, inhibit Kir6.1/SUR2B with IC_50_ values ranging from approximately 100 nM to 1 µM and exhibit no apparent inhibitory activity toward Kir6.2/SUR1. Functional analysis of heterologously expressed subunit combinations of Kir6.1, Kir6.2, SUR1, SUR2A, and SUR2B and demonstrated that all four inhibitors act on SUR2 to induce channel inhibition. Interestingly, VU0543336 and VU0212387 exhibit paradoxical stimulatory effects on Kir6.2/SUR1 at higher doses. This study broadens our understanding of K_ATP_ channel pharmacology, generally, and reveals novel chemical matter for the development of Kir6.1/SUR2-selective drugs, specifically.

## Introduction

K_ATP_ channels are regulated by intracellular ATP and ADP and thereby link cellular metabolism with cell membrane excitability [1–3]. K_ATP_ channels are hetero-octamers formed by the assembly of four pore-forming inward rectifier potassium (Kir) channel subunits (Kir6.1 or Kir6.2) and four regulatory sulphonylurea receptor (SUR) subunits (SUR1, SUR2A, or SUR2B) from the ABC transporter family [4]. Kir6.1 and Kir6.2 are encoded by *KCNJ8* and *KCNJ11*, respectively, SUR1 is encoded by *ABCC8*, and SUR2A and SUR2B are splice variants of *ABCC9* [4–7]. The molecular diversity of the K_ATP_ channel family is expanded by the cell type-specific expression, assembly, and function of different Kir6 and SUR subunit combinations exhibiting unique physiological, regulatory, and pharmacological properties [8–12]. Kir6.2/SUR1 is the major isoform expressed in the brain and insulin-secreting beta cells of the pancreas [13]. Inhibitory sulfonylurea drugs, such as glibenclamide, have been used clinically for many decades to promote insulin secretion and lower blood sugar in patients with type 2 diabetes [12,14]. The major K_ATP_ channel subtype expressed in vascular smooth muscle cells is Kir6.1/SUR2B, which functions to regulate vascular tone and blood pressure [2,15–19]. The Kir6.1/SUR2-specific activator drug, pinacidil, was once used to treat severe hypertension [20]; however, this drug was discontinued by the FDA due to untoward side effects such as headache and edema [21]. Furthermore, there is emerging evidence that highlights the critical need for developing specific inhibitors of vascular K_ATP_ channels for the treatment of diverse vascular-related disorders, such as Cantu syndrome, a rare genetic disease resulting from gain-of-function mutations in *KCNJ8* or *ABCC9* [22–27], patent ductus arteriosus [28,29], and sepsis [30].

A major barrier to developing therapies for these and other vascular disorders is a lack of specific Kir6.1/SUR2B inhibitors. All clinically used K_ATP_ channel inhibitors were developed for the treatment of type 2 diabetes and thus exhibit preferential or equivalent activity toward Kir6.2/SUR1 [14]. The drug, glibenclamide, for example, inhibits both Kir6.2/SUR1 and Kir6.1/SUR2B with approximately equal potency and therefore cannot be used to treat vascular disorders due to its risk of also inducing hypoglycemia [12,14]. Thus, new classes of inhibitors that are selective for Kir6.1/SUR2B over Kir6.2/SUR1 are critically needed for exploring the therapeutic potential of vascular K_ATP_ channels.

In an effort to identify new inhibitors, we recently screened 47,872 chemically diverse small molecules from the Vanderbilt Institute of Chemical Biology Discovery Collection. The high-throughput screen employed a fluorescence-based, quantitative thallium flux assay that we have used for a variety of potassium channels [31–37]. Verified hits from the primary screen were then counter screened against Kir6.2/SUR1 to identify inhibitors that are specific for Kir6.1/SUR2B. The most potent inhibitor identified from the screen was a compound named VU0542270, which we reported on recently [29]. VU0542270 inhibits Kir6.1/SUR2B with an IC_50_ of approximately 100 nM and has no apparent activity toward Kir6.2/SUR1 or several other members of the Kir channel family. The selectivity of VU0542270 is achieved through binding to SUR2. Importantly, VU0542270 induces contraction of ductus arteriosus vessels with the same potency of the nonspecific K_ATP_ channel inhibitor, glibenclamide. This work provides a key proof-of-concept that vascular-specific K_ATP_ channel inhibitors can be discovered using a molecular target-based HTS approach. Here, we expand on these findings by reporting the structures and basic pharmacological properties of four additional potent and selective Kir6.1/SUR2B inhibitors that are chemically distinct from VU0542270. These compounds are named VU0605768, VU0212387, VU0543336, and VU0544086.

## Methods

### Chemicals

VU0212387 (F3226–2876), VU0543336 (F6279–0409), VU0605768 (F3226–2876), and VU0544086 (F6295–1075) were purchased from Life Chemicals. VU0542270 was synthesized locally at the Warren Center for Neuroscience Drug Discovery using methods described previously [29]).

### Heterologous expression of K_ATP_ channels

The establishment of stably transfected tetracycline-inducible expression systems in human embryonic kidney-293 (T-Rex-HEK293)(Thermo Fisher Scientific) cells for the expression of Kir6.1/SUR2B and Kir6.2/SUR1 was detailed in earlier work [29,34]. Briefly, T-Rex-HEK293 cells underwent co-transfection with pcDNA5/TO-Kir6.1 or pcDNA5/TO-Kir6.2 (inducible expression) and pcDNA3.1-SUR2B or pcDNA3.1-SUR1 (constitutive expression) using Lipofectamine LTX following the manufacturer’s instructions and were then subjected to selection with antibiotics. Monoclonal cell lines were established through limiting dilution and characterization in thallium flux assays described below. Cells were cultured in Dulbecco’s modified Eagle’s medium (DMEM; Gibco 11,965–092) supplemented with heat-inactivated FBS (bio-techne, Minneapolis, MN S11150H,10%), Blasticidine HCl (Gibco, A11139–03, 10 µg/mL), Penicillin–Streptomycin (Gibco 15,140–122, 2 mM), G418 Sulfate (CORNING, Corning, NY, 30–234-CR, 1 mg/mL), and Hygromycin B (Invitrogen, Carlsbad, CA 10,687–010, 250 µg/mL). K_ATP_ channel selectivity assays were performed in HEK293 cells transiently transfected with the indicated subunit using Lipofectamine LTX. The cells were incubated for 48 h after transfection before performing thallium flux assays.

### Quantitative thallium flux assays

Thallium flux assays were conducted using the established methods [29]. Stably transfected T-REx-HEK-293 cells expressing Kir6.2/SUR1 or Kir6.1/SUR2B were cultured overnight in polyamine-coated plates (black-walled, clear-bottomed; BD, Bedford, MA) at a density of 20,000 cells in 20 µl per well of cell culture media. The expression of Kir6.2/SUR1 or Kir6.1/SUR2B channels was induced by treating cells with 1 µg/ml tetracycline overnight. The following day, cells were rinsed with an assay buffer (Hanks’ Balanced Salt Solution supplied with 20 mM HEPES), loaded with the thallium-sensor dye Brilliant Thallos-AM AM (Ion Bioscience, San Marcos, TX) for 1 h, and then washed again with assay buffer to remove unincorporated dye. The cell plate was subsequently placed in a Panoptic Kinetic Imaging Plate Reader (Wavefront Bioscience, Franklin, TN) and imaged at a frequency of 1 Hz using 482/35 nm (excitation) and 536/40 nm (emission) filter sets. Kir6.1/SUR2B channels were activated with 10 µM pinacidil, whereas Kir6.1/SUR1 was activated with 30 µM VU0071063 [34]. To activate K_ATP_ channels independent of activators with ATP-depletion method, cells were treated with a cocktail of metabolic inhibitors (2.5 mM sodium cyanide and 20 mM 2-deoxy glucose) for 25 min before imagine [38]. Control and test compounds diluted freshly from DMSO stocks into assay buffer were added to wells for four minutes before adding 0.5 mM chloride-free thallium stimulus buffer (Ion Bioscience) to initiate thallium flux through the expressed K_ATP_ channels. Thallium flux was recorded from all 384-wells for 2 min before terminating the experiment. Control compounds used were pinacidil (10 µM; SUR2-specific activator), VU0071063 (10 µM; SUR1-specific activator) [39], and glibenclamide (10 µM; nonselective K_ATP_ inhibitor). Selectivity assays against other Kir channels were performed as described previously [31,32].

### Whole-cell patch clamp electrophysiology

Whole-cell patch clamp electrophysiology experiments were conducted as described previously [29]. Briefly, stably transfected T-Rex-HEK293 cells expressing Kir6.1/SUR2B were seeded at a density of 250,000 cells per 35-mm dish coated with tissue culture-treated polystyrene and incubated at 37°C in a 5% CO2 atmosphere. Whole-cell patch-clamp experiments were initiated 48 h post-seeding. On the day of the experiment, cells were dissociated and seeded onto poly-L-lysine-coated glass coverslips, where they were allowed to recover for at least one hour in a 37°C/5% CO_2_ incubator prior to the recordings. Patch pipettes with resistances of 2 to 3 MΩ were employed, containing an internal solution composed of 130 mM KCl, 2 mM MgCl_2_, 1 mM EGTA, 20 mM HEPES-free acid, and 1 mM Na_2_ADP, adjusted to pH 7.3 with KOH and an osmolarity of 275 mOsmol/kg with sucrose. The extracellular solution consisted of 135 mM NaCl, 5 mM KCl, 2 mM CaCl_2_, 1 mM MgCl_2_, 5 mM glucose, and 10 mM HEPES-free acid adjusted to pH 7.4 with NaOH. Test compounds were initially diluted in DMSO before being further diluted into the bath solution, maintaining a final DMSO concentration of less than 0.1% v/v. Recordings of macroscopic currents were conducted in a voltage-clamp setup using an Axopatch 200B Amplifier (Molecular Devices, Sunnyvale, CA), with data sampled at 5 kHz and low-pass filtered at 1 kHz. A ramp protocol was used to generate current–voltage curve relationships. The voltage protocol involved holding the cells at −75 mV, followed by a 200-msec step to −120 mV, a linear ramp to 120 mV over 240 msec at a rate of 1.2 mV/msec, maintaining at 120 mV for 15 msec, and finally stepping back to −120 mV for 100 msec, with this sequence repeated every 5 seconds. The experiments were concluded by applying a 10 µM concentration of the nonselective K_ATP_ channel blocker, glibenclamide, to confirm completeness of channel activity and to quantify leak current. Only cells demonstrating greater than 90% inhibition with glibenclamide were included in data analysis. All data acquisition and analyses were performed using pClamp software version 9.2 (Molecular Devices).

### Data analysis and statistics

The kinetic fluorescence measurements (F) from each well were normalized to the initial reading (F_0_), producing static ratios (F/F_0_) that adjust for variations in cell count and dye uptake. Baseline (min) and maximum(max) values of these static ratios were recorded at 250 s and 295 s, respectively. The response (maxmin2) was determined by subtracting the min value from the max value. Percent inhibition was calculated by normalizing each well’s response to the positive and negative controls. Concentration–response curves were fitted using a single-site, four-parameter logistic function in Prism version 4.0 (GraphPad Software Inc., San Diego, CA) to calculate the half-maximal inhibitory concentration (IC_50_). Statistical comparisons of IC_50_ values were performed using an extra sum-of-squares F test and the Mann–Whitney test. A p-value of less than 0.05 was considered statistically significant.

## Results

### VU0212387, VU0543336, VU0605768, and VU0544086 are potent Kir6.1/SUR2B inhibitors

**Figure 1.**
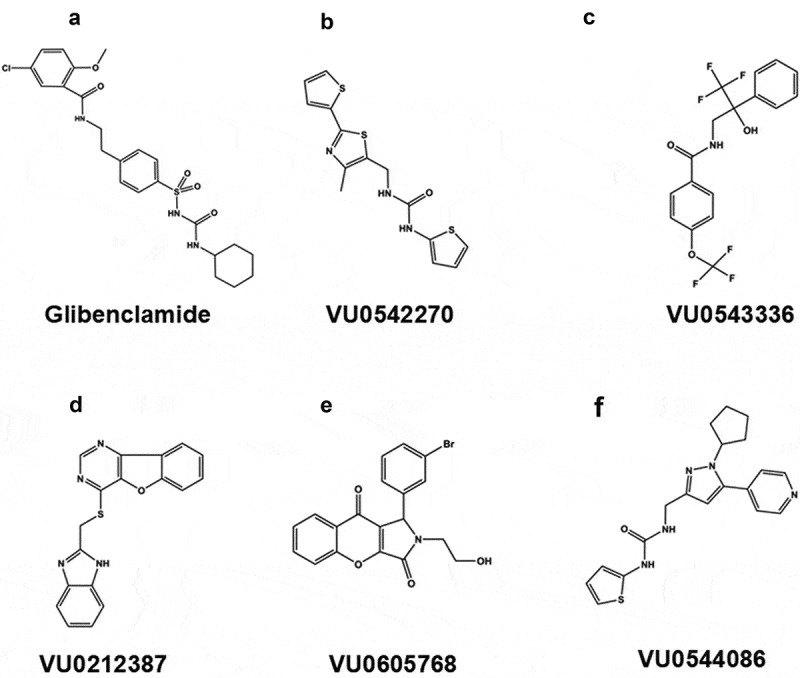
Chemical structures of novel Kir6.1/SUR2B inhibitors.

Hits from a primary screen of 47,872 compounds against Kir6.1/SUR2B were subsequently counter screened against Kir6.2/SUR1 to eliminate all nonspecific K_ATP_ channel inhibitors from further study. Concentration–response curves (CRCs) were then prioritized for 99 compounds exhibiting at least 92% inhibition at a single dose of 10 µM. As noted above and published previously [29], the most potent inhibitor was VU0542270. The next four most potent compounds were VU0212387 (4-(((1 H-benzo[d]imidazol-2-yl)methyl)thio)benzofuro[3,2-d]pyrimidine), VU0543336 (*N*-(3,3,3-trifluoro-2-hydroxy-2-phenylpropyl)-4-(trifluoromethoxy)benzamide), VU0605768 (1-(3-bromophenyl)-2-(2-hydroxyethyl)-1,2-dihydrochromeno[2,3-c]pyrrole-3,9-dione), and VU0544086 (1-((1-cyclopentyl-5-(pyridin-4-yl)-1 H-pyrazol-3-yl)methyl)-3-(thiophen-2-yl)urea). Their chemical structures are shown in Figure 1.

**Figure 2.**
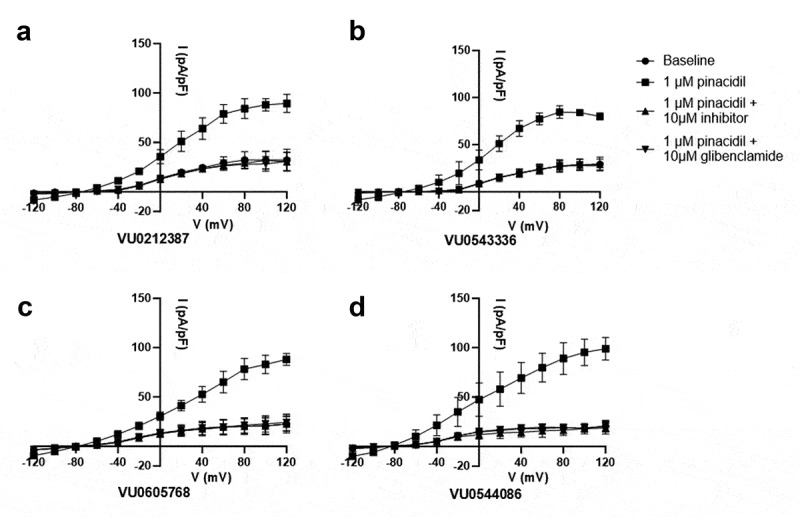
Confirmation of VU0212387, VU0543336, VU0605768, or VU0544086-dependent inhibition of Kir6.1/SUR2B with patch clamp electrophysiology. IV relationships were measured from T-Rex-HEK293-Kir6.1/SUR2B cells bathed in control buffer (circles), 1 μM pinacidil (squares), 1 μM pinacidil + 10 μM VU compound (upright triangle), or 1 μM pinacidil + 10 μM glibenclamide (upside-down triangle). VU compounds tested were (a) VU0212387, (b) VU0543336, (c) VU0605768, or (d) VU0544086. Data mean ± SD current density (n = 3).

Gold-standard voltage-clamp electrophysiology was used to confirm the inhibitory effects of VU0212387, VU0543336, VU0605768, and VU0544086 on Kir6.1/SUR2B channel activity (Figure 2a–d). As reported previously for this stably transfected cell line (29), whole-cell currents were small in control buffer but increased significantly upon bath addition of pinacidil (data not shown). Subsequent addition of 10 µM VU0212387, VU0543336, VU0605768, or VU0544086 in the continued presence of pinacidil led to complete K_ATP_ channel inhibition.

**Figure 3.**
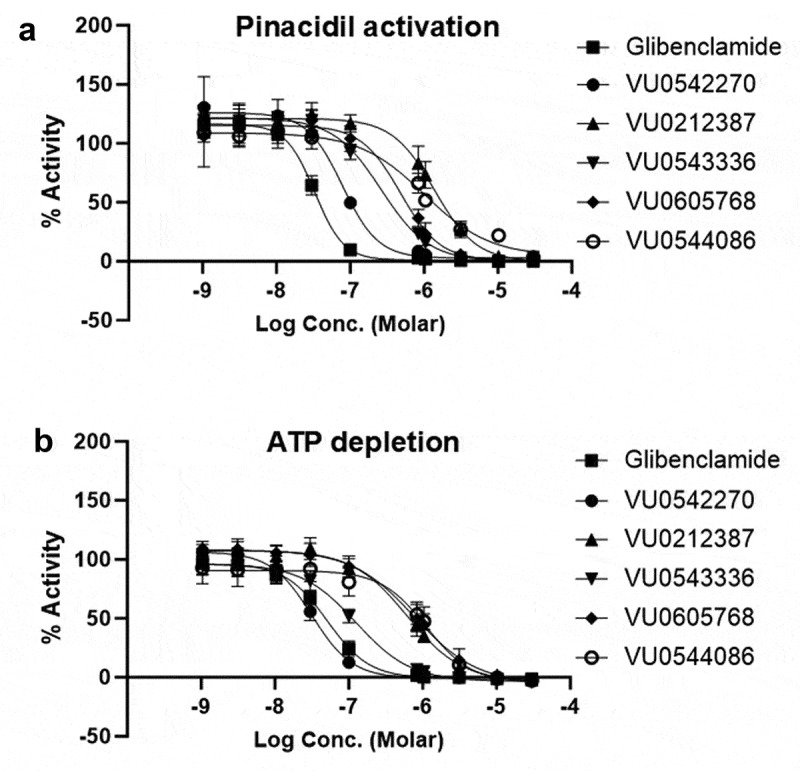
Dose–response curves for Kir6.1/SUR2B inhibitors. Stably transfected T-Rex-HEK293 cells expressing Kir6.1/SUR2B were treated with escalating doses ofVU0212387, VU0543336, VU0605768, VU0544086, VU0542270, or glibenclamide (a) in the presence of 10 μM pinacidil or (b) after ATP depletion with sodium cyanide and 2-deoxy-glucose. Mean % inhibition was fitted with a four-parameter logistic. The derived IC50 values and 95% confidence intervals (CI) were (a) VU0543336 = 0.271 μM [216,319], VU0212387 = 1.4 μM [1.2-1.6], VU0605768 = 0.479 μM [335-750], VU0544086 = 1 μM [0.8-1.6], VU0542270 = 0.078 μM [66-93], glibenclamide = 0.033 μM [30-37]; (b) VU0543336 = 0.126 μM [109-146], VU0212387 = 0.619 μM [543-698], VU0605768 = 0.779 μM [642-937], VU0544086 = 1.0 μM [0.9-1.3], VU0542270 = 0.031 μM [29-34], glibenclamide = 0.051 μM [46-59].

Ten-point, threefold dilution, and dose-response experiments were performed in quantitative thallium flux experiments against Kir6.1/SUR2B with doses ranging from 30 µM to 1 nM. Channels were activated with either metabolic poisoning cocktail or 10 µM pinacidil. Glibenclamide and VU0542270 were used as control inhibitors. CRC data were fitted with 4-parameter logistic functions to derive IC_50_ values for each compound. The resulting data are shown in Figure 3a (pinacidil activation) and Figure 3b (ATP depletion/metabolic poisoning activation). Glibenclamide, VU0542270, VU0212387, VU0543336, VU0605768, and VU0544086 inhibited Kir6.1/SUR2B under both activation conditions in a dose-dependent manner (Figure 3) with distinct IC_50_ values (Table 1). The IC_50_ of VU0542270 was significantly lower than that of glibenclamide (extra sum-of-squares F test, *p* < 0.0001; Mann–Whitney test, *p* < 0.001) in the ATP depletion condition.

**Table 1.** Inhibitor potency determined in thallium flux assays. Experiments were performed in thallium flux assays. Data are mean IC50 values (in µM) derived from curve fitting to CRC data. See text for details.

	Kir6.1/SUR2B (µM)		Kir6.2/SUR1 (µM)	
Inhibitor	Pinacidil activation	ATP depletion	VU0071063 activation	ATP depletion
VU0543336	0.3	0.1	>30	>30
VU0212387	1.4	0.6	>30	>30
VU0605768	0.5	0.5	>30	>30
VU0544086	1.0	1.0	>30	>30
VU0542270	0.1	0.03	>30	>30
Glibenclamide	0.03	0.05	0.03	0.1

### VU0212387, VU0543336, VU0605768, and VU0544086 do not inhibit Kir6.2/SUR1

**Figure 4.**
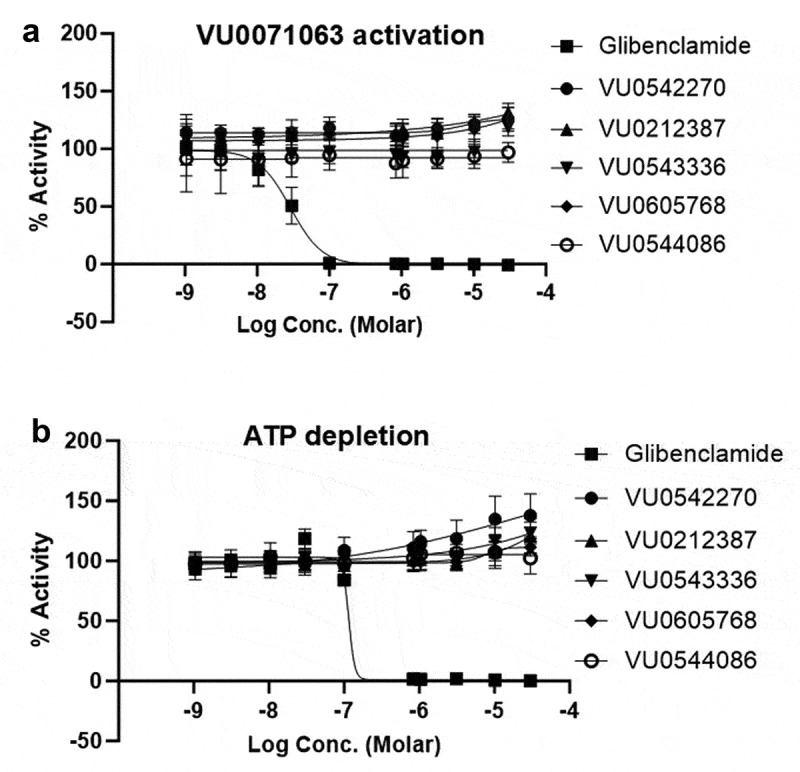
Dose-response curves of VU0543336, VU0212387, VU0605768, and VU0544086 against Kir6.2/SUR1. Stably transfected T-Rex-HEK293 cells expressing Kir6.2/SUR1 were treated with escalating doses of VU0543336, VU0212387, VU0605768, VU0544086, VU0542270, or glibenclamide (a) in the presence of 10 uM VU0071063 or (b) after ATP depletion with sodium cyanide and 2-deoxy-glucose. Average % inhibition was fitted with a four-parameter logistic. The derived IC50 values and 95% CI were (a) VU0543336 no fit, VU0212387 no fit, VU0605768 no fit, VU0544086 no fit, VU0542270 no fit, glibenclamide = 0.029 μM [22-36]; (b) VU0543336 no fit, VU0212387 no fit, VU0605768 no fit, VU0544086 no fit, VU0542270 no fit, glibenclamide = 100nM [NA].

The selectivity of glibenclamide, VU0542270, VU0212387, VU0543336, VU0605768, and VU0544086 were evaluated in similar dose–response experiments against Kir6.2/SUR1 channels activated with either VU0071063 (Figure 4a) or ATP depletion (Figure 4b). As expected, glibenclamide inhibited Kir6.2/SUR1 under both activating conditions, whereas VU0542270, VU0212387, VU0543336, VU0605768, and VU0544086 had no effect on Kir6.2/SUR1 under either condition across the full range of doses tested. IC_50_ values are summarized in Table 1.

### VU0212387, VU0543336, VU0605768, and VU0544086 are SUR2-specific inhibitors

**Figure 5.**
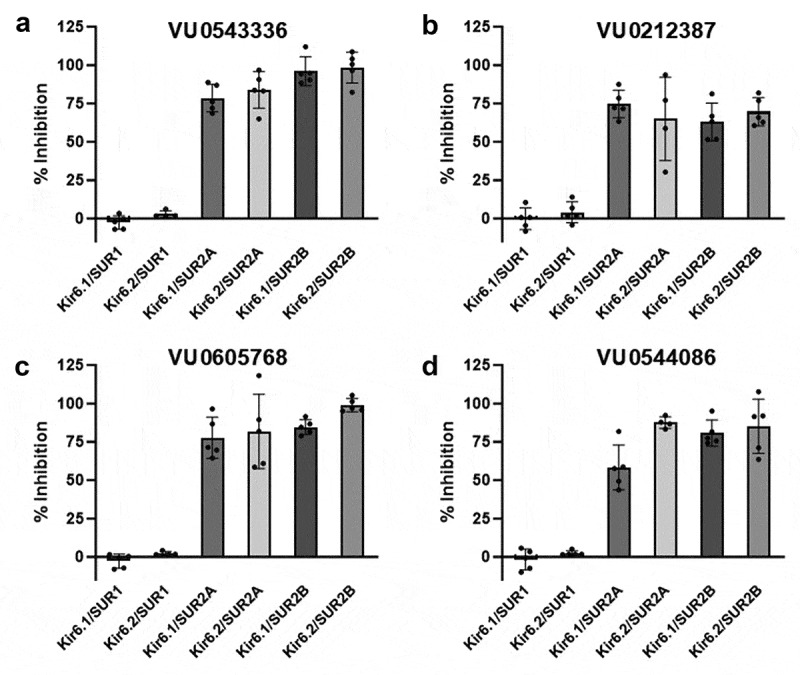
Inhibitor SUR2 vs SUR1 selectivity. Mean ± S.D. percent inhibition of the indicated KATP isoforms with (a) VU0543336, (b) VU0212387, (c) VU0605768, or (d) VU0544086. HEK293 cells transiently transfected with plasmids encoding Kir6.1, Kir6.2, SUR1, SUR2A, and/or SUR2B were treated with 3 μM inhibitors in the presence of 10 μM VU0071063 (SUR1) or pinacidil (SUR2) and evaluated in thallium flux assays.

To begin elucidating the binding site location of VU0212387, VU0543336, VU0605768, and VU0544086, we evaluated the activity of the inhibitors against K_ATP_ channels composed of different Kir and SUR subunit combinations expressed in HEK293 cells. Specifically, the compounds were tested against Kir6.1/SUR1, Kir6.2/SUR1, Kir6.1/SUR2A, Kir6.2/SUR2A, Kir6.1/SUR2B, and Kir6.2/SUR2B at a dose of 3 µM. As summarized in Figure 5, none of the compounds inhibited channel combinations containing SUR1, whereas all of the compounds inhibited K_ATP_ channels containing either SUR2A or SUR2B. We expanded the selectivity analysis to include 10 other members of the Kir channel family. With the exception VU0543336 and VU0212387, which inhibit Kir4.2 with IC_50_ values of 12 µM and 17 µM, respectively, VU0544086, which inhibits Kir3.1/3.4 with an IC_50_ of 15 µM, and VU0212387, which inhibits Kir7.1 with an IC of 12 µM, the ancillary pharmacology of the four compounds was relatively clean (Table 2).

**Table 2.** Inhibitor selectivity within the Kir channel family. Experiments were performed in thallium flux assays. Data are mean IC50 values (in µM) determined from fits to CRC data. Percent maximal inhibition observed at 30 µM is shown in parentheses. *22.8% activation at 30 µM. See text for details.

Channel	VU0543336 (μM)	VU0212387 (μM)	VU0605768 (μM)	VU0544086 (μM)
Kir1.1	>30	>30	>30	>30
Kir2.1	>30	>30	>30	>30
Kir2.2	>30	>30	>30	>30
Kir2.3	>30	>30	>30	>30
Kir3.1/3.2	>30	>30	>30	>30
Kir3.1/3.4	>30*	>30	>30	16.2 (92)
Kir4.1	>30	>30	>30	>30
Kir4.2	11.8 (110)	17.1 (56)	>30	>30
Kir4.1/5.1	>30	>30	>30	>30
Kir7.1 (M125R)	>30	12.3 (69)	>30	>30

### VU0212387, VU0543336, and VU0605768 activate Kir6.2/SUR1 at high doses

**Figure 6.**
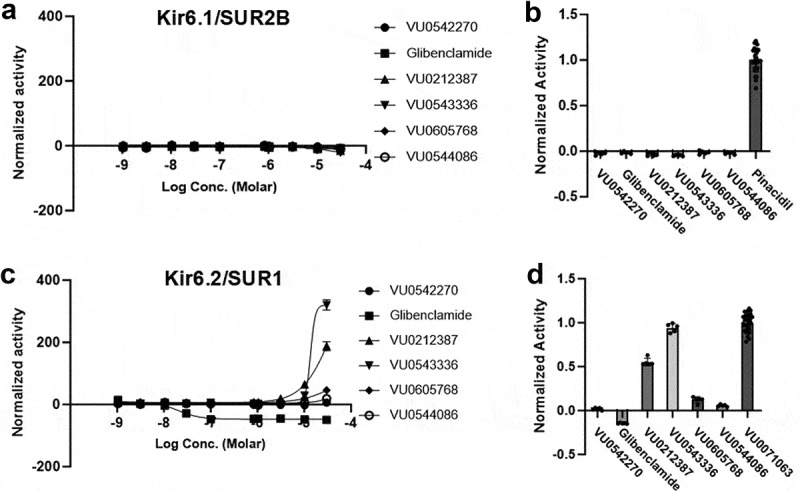
Paradoxical activation of Kir6.2/SUR1. Stably transfected T-Rex-HEK293 cells expressing Kir6.1/SUR2B or Kir6.2/SUR1 were treated with escalating doses of VU0543336, VU0212387, VU0605768, VU0544086, VU0542270, or glibenclamide. Fluorescence intensity at each dose was normalized to that in the absence of drug. (a) All 6 compounds failed to activate Kir6.1/SUR2B. (b) Activities of indicated compounds at 30 μM toward Kir6.1/SUR2B normalized to that of 30 μM pinacidil. (c) VU0543336, VU0212387, and VU0605768 induced dose-dependent Kir6.2/SUR1 activation, whereas VU0071063, VU0542270, and glibenclamide did not. (d) Activities of indicated compounds at 30 μM toward Kir6.2/SUR1 normalized to that of 30 μM VU0071063.

As shown in Figure 4, some compounds appeared to activate Kir6.2/SUR1 at higher doses under both pharmacological and metabolic inhibition conditions. We therefore systematically explored this possibility in dose–response experiments against Kir6.2/SUR1 in the absence of channel pre-activation. Kir6.1/SUR2B was also evaluated for comparison. Glibenclamide, VU0542270, VU0212387, VU0543336, VU0605768, and VU0544086 failed to activate Kir6.1/SUR2B at doses up to 30 µM (Figure 6a–b). Similarly, glibenclamide, VU0542270, or VU0544086 failed to activate Kir6.2/SUR1. However, the other three compounds dose-dependently activated Kir6.2/SUR1 with a rank-order efficacy of VU0543336 > VU0212387 >> VU0605768 (Figure 6c–d). EC_50_ values could not be determined from these data.

## Discussion

The field of K_ATP_ channel pharmacology began in the 1940s with the serendipitous discovery that sulfonamide drugs used for treating typhoid fever also induced hypoglycemia due to stimulation of insulin secretion from the pancreas [40]. We now understand that sulfonamide-containing drugs stimulate insulin secretion by inhibiting pancreatic Kir6.2/SUR1 channels by binding to the SUR1 subunit of the K_ATP_ channel complex [41]. This well-known pharmacology underlies the clinical efficacy of sulfonylurea drugs such as glibenclamide in managing glycemic levels in type 2 diabetics. The growing prevalence of type 2 diabetes has been a major driving force in the pharmaceutical industry for creating drugs that are highly selective for SUR1 over SUR2, leaving the state of SUR2 pharmacology virtually undeveloped.

Emerging evidence highlights the important role of vascular K_ATP_ channels in a spectrum of vascular disorders. Kir6.1/SUR2B channels are expressed in ductus arteriosus (DA) tissues and have been identified as a promising therapeutic target for the management of patent ductus arteriosus (PDA) [28,29]. This is supported by the observation that *KCNJ8* and *ABCC9* transcripts are enriched in DA tissues compared to other vessels [28,42,43]. Furthermore, more than half of the patients with Cantu syndrome, a condition characterized by gain-of-function mutations in *KCNJ8* or *ABCC9*, present with symptomatic PDA at birth [12]. Colin Nichols and colleagues have generated several mouse models carrying different Cantu syndrome mutations that should be useful in evaluating the therapeutic efficacy of VU inhibitors in treating this complex disease [12]. Additionally, Kir6.1 and SUR2B transcript levels are also found to be up-regulated in endotoxic rats [44]. Compelling evidence from in vivo experiments demonstrates the efficacy of glibenclamide in rapidly reversing hypotension in these animals, implicating the hyperactivity of Kir6.1/SUR2B channels in the pathogenesis of sepsis-induced hypotension [45]. Furthermore, the Kir6.1/SUR2B channel opener levcromakalim has been identified as a potent trigger of migraine headaches, acting through the dilation of extracerebral arteries [45,46]. These findings collectively highlight the critical involvement of Kir6.1/SUR2B channels in various vascular pathologies and underscore the pressing need for the development of vascular-specific KATP inhibitors.

The dearth of SUR2 pharmacology and growing appreciation that Kir6.1/SUR2B channels represent putative drug targets for these vascular-related disorders led us to take a molecular target-based approach to developing the Kir6.1/SUR2B pharmacology. Screening approximately 48,000 chemically diverse compounds led to the discovery of dozens of inhibitors that are selective for Kir6.1/SUR2B over Kir6.2/SUR1. The most potent of these is VU0542270, which we reported on recently [29]. VU0542270 inhibits Kir6.1/SUR2B with an IC_50_ of approximately 100 nM, is highly selective for SUR2-containing K_ATP_ channels, and induces vasoconstriction of isolated mouse ductus arteriosus vessels with the same potency as glibenclamide. These properties make VU0542270 the current state-of-the art in vascular K_ATP_ channel inhibitors [29].

In the present work, we report the chemical structures and basic pharmacological properties of four additional Kir6.1/SUR2B inhibitors identified in our screen, with the goal of stimulating vigorous research activities in the broader vascular K_ATP_ channel field. Estimated IC_50_ values of the compounds range from approximately 0.1–1.4 µM for inhibition of Kir6.1/SUR2B. All four compounds inhibit Kir6.1/SUR2B channels that have been activated either pharmacologically with pinacidil or with ATP depletion, confirming that inhibition does not simply reflect the drug-induced displacement of pinacidil from its binding site in SUR2. The off-target activity of the compounds within the Kir channel family is relatively clean, with a few notable exceptions worth mentioning. VU0543336 and VU0212387 inhibit Kir4.2 with IC_50_s of 12 µM and 17 µM, respectively. Kir4.2 is an emerging drug target of interest due to its important roles in solute transport in the kidney proximal tubule [47]. The only other published Kir4.2 inhibitor is VU0134992 (IC_50_ = 8.2 µM); however, this compound also inhibits Kir3.1/3.2 (IC_50_ = 2.5 µM), Kir3.1/3.4 (IC_50_ = 3.1 µM), and Kir4.1 (IC_50_ = 5.2 µM) with greater than 90% efficacy at 30 µM [32]. With their apparently clean ancillary pharmacology, VU0543336 and VU0212387 might represent useful in vitro or ex vivo tool compounds for probing the physiology of Kir4.2 in the proximal tubule. It should also be noted that VU0544086 inhibits Kir3.1/3.4 channels with an IC_50_ of 15 µM. Because Kir3.1/3.4 channels carry the acetylcholine-activated Kir current (I_KACh_) in the atria of the heart, dual inhibition of vascular Kir6.1/SUR2B and I_KACh_ might lead to confounding effects on cardiovascular physiology if VU0544086 administered in vivo. The only other off-target activity observed in the present study was the inhibition of Kir7.1 by VU0212387 (IC_50_ = 12 µM). Kir7.1 plays important roles in regulating melanocortin signaling in the brain [48], retinal pigmented epithelial function in the eye [49], and possibly electrolyte transport in the kidney [50]. Potential off-target effects of VU0212387 on these organ systems should be considered when under study.

In our view, one of the most exciting outcomes of this work so far is the discovery of structurally diverse inhibitors of SUR2-containing K_ATP_ channels, for at least two reasons. First, this shows a critical proof-of-concept that potent and specific Kir6.1/SUR2B inhibitors can be identified using molecular target-based high-throughput screening of publicly available small-molecule libraries. Secondly, VU0542270, VU0212387, VU0543336, VU0605768, and VU0544086 all represent chemical scaffolds that are distinct not only from each other but also from all known sulfonylurea drugs that preferentially inhibit SUR1. Most of the publicly disclosed drug discovery efforts to develop improved anti-diabetic medications focused on compounds that contain a sulfonamide group. Some of the drugs that have been used clinically to treat type 2 diabetes, such as tolbutamide and glibenclamide, also exhibit cross-inhibition of SUR2-containing K_ATP_ channels [14]. The recent determinations of high resolution Kir6.2/SUR1 and Kir6.1/SUR2B structures in complex with glibenclamide revealed that this drug interacts with highly conserved regions of SUR1 and SUR2B, in part, through sulfonamide interactions with the channel protein. The discovery of several distinct structural classes of SUR2-selective inhibitors lacking sulfonamide groups raises the possibility that these compounds inhibit Kir6.1/SUR2B through distinct binding sites and/or modes of action. We anticipate that structural biology, computational homology modeling, and structure-based drug design approaches will enable the development of next-generation inhibitors of vascular K_ATP_ channels that are useful for preclinical in vivo studies.

An unexpected outcome of this study was the discovery that VU0543336, VU0212387, and VU0605768 activate Kir6.2/SUR1 at higher drug doses. This highlights the complexity of K_ATP_ channel pharmacology and suggests a possible dual nature of drug interactions with these channel proteins. Unlike the expansive toolkit of Kir6.2/SUR1 channel inhibitors available, there are comparatively few channel activators. Congenital hyperinsulinism (CHI) is a genetic disorder caused by loss-of-function mutations in either SUR1 or Kir6.2 [51]. Diazoxide is a sulfonyl group-containing drug used clinically to stimulate residual Kir6.2/SUR1 in CHI patients [52]. Unfortunately, however, diazoxide also exhibits cross activation of SUR2-containing channels, which limits its clinical utility. It is conceivable that VU0543336, VU0212387, and VU0605768 could be used as starting chemical scaffolds for developing SUR1-specific activators for treating CHI.

In conclusion, the discovery of structurally diverse vascular K_ATP_ channel inhibitors represents an important step in exploring the therapeutic potential of K_ATP_ channels in the treatment of diverse cardiovascular disorders. Next steps include understanding inhibitor mechanism of action using structural, computational, and mutagenesis strategies, optimization of inhibitor potency and selectivity with medicinal chemistry, evaluating drug metabolism and pharmacokinetic properties of inhibitor analogs, and testing optimized inhibitors in various pre-clinical animal models of human diseases/disorders, such as patent ductus arteriosus, Cantu syndrome, and septic shock. We anticipate that this work will establish a comprehensive pharmacological toolkit for rigorously testing the value of targeting Kir6.1/SUR2B channels for improving human health.

## Data Availability

Primary screening data not included in the article may be made available upon request.
